# Treating the Side Effects of Exogenous Glucocorticoids; Can We Separate the *Good* From the *Bad*?

**DOI:** 10.1210/endrev/bnad016

**Published:** 2023-05-30

**Authors:** Riccardo Pofi, Giorgio Caratti, David W Ray, Jeremy W Tomlinson

**Affiliations:** Oxford Centre for Diabetes, Endocrinology and Metabolism, NIHR Oxford Biomedical Research Centre, University of Oxford, Churchill Hospital, Oxford OX3 7LE, UK; Oxford Centre for Diabetes, Endocrinology and Metabolism, NIHR Oxford Biomedical Research Centre, University of Oxford, Churchill Hospital, Oxford OX3 7LE, UK; Oxford Centre for Diabetes, Endocrinology and Metabolism, NIHR Oxford Biomedical Research Centre, University of Oxford, Churchill Hospital, Oxford OX3 7LE, UK; NIHR Oxford Biomedical Research Centre, John Radcliffe Hospital, Oxford OX3 9DU, UK; Oxford Kavli Centre for Nanoscience Discovery, University of Oxford, Oxford OX37LE, UK; Oxford Centre for Diabetes, Endocrinology and Metabolism, NIHR Oxford Biomedical Research Centre, University of Oxford, Churchill Hospital, Oxford OX3 7LE, UK

**Keywords:** steroid hormones, adverse effects, 11β-hydroxysteroid dehydrogenase type 1 inhibition, SEGRA, chronopharmacology

## Abstract

It is estimated that 2% to 3% of the population are currently prescribed systemic or topical glucocorticoid treatment. The potent anti-inflammatory action of glucocorticoids to deliver therapeutic benefit is not in doubt. However, the side effects associated with their use, including central weight gain, hypertension, insulin resistance, type 2 diabetes (T2D), and osteoporosis, often collectively termed *iatrogenic Cushing's syndrome*, are associated with a significant health and economic burden. The precise cellular mechanisms underpinning the differential action of glucocorticoids to drive the desirable and undesirable effects are still not completely understood. Faced with the unmet clinical need to limit glucocorticoid-induced adverse effects alongside ensuring the preservation of anti-inflammatory actions, several strategies have been pursued. The coprescription of existing licensed drugs to treat incident adverse effects can be effective, but data examining the prevention of adverse effects are limited. Novel selective glucocorticoid receptor agonists and selective glucocorticoid receptor modulators have been designed that aim to specifically and selectively activate anti-inflammatory responses based upon their interaction with the glucocorticoid receptor. Several of these compounds are currently in clinical trials to evaluate their efficacy. More recently, strategies exploiting tissue-specific glucocorticoid metabolism through the isoforms of 11β-hydroxysteroid dehydrogenase has shown early potential, although data from clinical trials are limited. The aim of any treatment is to maximize benefit while minimizing risk, and within this review we define the adverse effect profile associated with glucocorticoid use and evaluate current and developing strategies that aim to limit side effects but preserve desirable therapeutic efficacy.

## Graphical Abstract

**Graphical Abstract:**
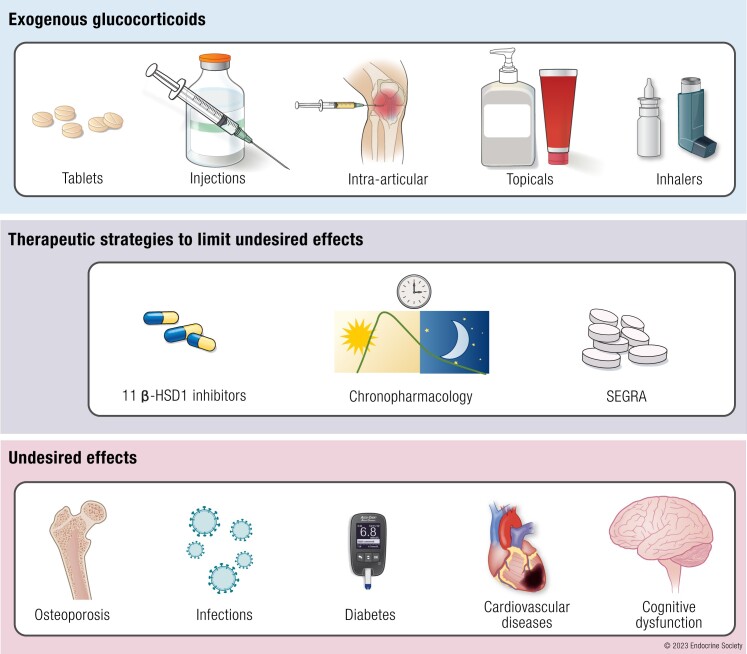

**Essential Points**

- Despite clinical awareness of the adverse effects of glucocorticoid treatment, its use has remained remarkably constant over many years: currently 1% to 3% of the worldwide population are prescribed glucocorticoid therapy and long-term prescriptions have increased by more than 30% over the past 20 years.
- All routes of glucocorticoid administration (topical, inhaled, intranasal, and intra-articular) are associated with the development of systemic adverse effects (notably affecting mortality, infection risk, and metabolic, cardiovascular, and bone health), but the precise contribution of dose and duration of treatment is difficult to establish.
- Glucocorticoids mediate their beneficial (anti-inflammatory) and undesired effects through classical (genomic) and nongenomic actions.
- Complementary approaches are currently being used to treat and limit the adverse effects of glucocorticoids including antihypertensive therapies and glucose-lowering medications as well as the use of bone antiresorptive therapy.
- Novel drugs are currently in development that aim to separate the negative side effects from the beneficial anti-inflammatory actions, including selective glucocorticoid receptor agonists and 11β-hydroxysteroid dehydrogenase type 1 inhibitors.

Currently, 1% to 3% percent of the worldwide population are prescribed glucocorticoid (GC) therapy (1-5). There are differences in the prevalence of GC prescriptions between countries, and this may reflect local prescribing and treatment practices as well as the methodology used to collate the data. Chronic use is reported in 0.5% to 1.8% of patients (6, 7), and the prevalence of use increases significantly with age (1, 4-9) (Table 1). Despite clinical awareness of the adverse effects of GC treatment, as well as the increased use of steroid sparing treatment regimens, the use of GC treatment has remained remarkably constant over many years; in fact, there is evidence to suggest that long-term prescriptions have increased by more than 30% over the past 20 years (5). Data from the UK national report on medicines use indicate that GCs are now among the top-10 prescribed drug by cost (third place overall) and account for approximately 6.4% of total public expenditure on medication (10). When examining data from these studies, there are a number of confounding issues and limitations that need to be considered. Most of the studies reported GC prescriptions, which may not relate directly to administration, and relate to primary care rather than prescriptions in a hospital setting.

**Table 1.** Summary of clinical studies estimating steroid prescription prevalence

References	Study population	GC type and use	GCs prescription	Sex/age prevalence	Most prescribed GCs
Einarsdottir et al, Clin End, 2020 (8)	Western Sweden population, 1,585,335 adults (56% W); (2007-2014)	Oral GCs (>5 mg of prednisolone) Short/mid/long-term users	Overall 14.1%. - Short-term users 7.5% - Mid-term users 1.8% - Long-term users 0.5%	Highest prevalence in men aged >80 (24.4%) Lowest prevalence in men aged 10-19 years (7.5%)	Betamethasone (53.8%) Prednisolone (45%)
Waljee et al, BMJ, 2017 (9)	US population (<64 years), 1,548,945 adults (51% W); (2012-2014)	Oral GCs Only short-term (<30 days) users	Overall 21.1%	Prescriptions increase with age	Methylprednisolone (47%)
Laugesen et al, BMJ Open 2017 (4)	All the Danish population; (1999-2015)	Oral/injected GCs Short/long-term users	Annual prevalence 3% Elderly, up to 10%	Increased prevalence in women (OR 1.11) and throughout age: Aged 40-64, OR 10*; Aged >80, OR 25*. *When compared to age below 19	Prednisolone (50%)
Laugensen et al, EJE 2019 (3)	Danish population, 926,314 GCs users (54% W); (1999-2014)	Oral/injected GCs	Annual prevalence 3% Reduced prescription (6%) from 1999 to 2014 (increased use of targeted treatments)	Higher prevalence in women. Aged 70-79, 7%; Aged >80, 11%;	Prednisolone (53%)
Fardet et al, Rheumatology, 2011 (5)	UK population > 4 million adults (59% W); (1989-2008)	Oral GCs Long-term (>3 months) users	Overall 8.5%; Increased prescription (34%) from 1989 to 2009	Lower prescription (0.08%) in men aged 18-29; Higher prescription (3%) in women aged >80.	Prednisolone (92%)
Overman et al, Arthritis Care Res, 2013 (2)	US population, 26,248 adults (53% W), (1999-2008)	Oral GCs Short- term users (35%) Long-term users (65%, GCs received >3 months)	- Overall 1.2%	Men aged >80 prevalence 3.5% Women aged 70-79 prevalence 2.7%	Prednisolone (77%)
Bénard-Laribière et al, BMJ Open 2017 (6)	French population 382,572 adults (58% W); (2007-2014)	Oral GCs Short-term users (1 prescription, 68%); Mid-term users (2-5 prescriptions, 30%); Long-term users (>6 prescriptions, 2%)	Overall 17.1%. - Short-term users 11.8% - Mid-term users 4.6% - Long-term users 0.7%	Higher prevalence in women aged 50-59 years (21.9%)	Prednisolone (16%)
Walsh et al, BMJ, 1996 (7)	UK population 65,786 adults (52% W); 1995	Oral GCs, long-term users (>3 months)	Overall 0.5%	Higher prevalence in women aged >55 years (1.7%)	Prednisolone (97%)
Van Staa et al, QJM, 2000 (1)	UK population, 244,235 GC-users; 1997	Oral GCs, Short-term users (78%) Long-term users (22%, GCs prescribed >6 months).	Overall 0.9%	No differences between sexes; Prescriptions increase with age: highest in patients aged 70-79 (2.5%); lowest in patients aged 20-29 (0.2%)	Prednisolone (91%)

Abbreviations: GC, glucocorticoid; OR, odds ratio; W women.

Since their discovery in the 1940s, GCs have become a mainstay of therapy for many endocrine and nonendocrine indications. In endocrine disease, prescribed GCs are used for the diagnosis of Cushing's syndrome (mainly dexamethasone), but, more importantly, they are crucial for the management of adrenal insufficiency (11) and congenital adrenal hyperplasia (CAH) (12). In cases of adrenal insufficiency, they are usually prescribed in 2 to 3 divided doses per day, with 50% to 66% given in the morning on awakening, aiming at physiological doses and patterns of administration as replacement therapy (12-18). Conversely, supraphysiological (and still occasionally reverse-circadian) doses are often needed to control patients with CAH. For nonendocrine indications, GCs are prescribed for their potent anti-inflammatory and immunosuppressive effects. Synthetic GCs are used to treat patients with a wide range of immunologic and inflammatory disorders, such as asthma, chronic obstructive pulmonary disease, chronic inflammatory bowel diseases, rheumatic diseases, and malignancies. Despite the numerous advances in medical therapies for inflammatory and malignant conditions, GCs are still widely used, reflecting their potent clinical impact and, in some circumstances, a lack of efficacious alternative therapeutic options.

All GCs (endogenous and synthetic) bind and activate the GC receptor (GR) (see Classical Steroid Hormone Action section) with differing potencies and have differing pharmacokinetic and pharmacodynamic properties. Many GCs, although not all, are able to activate the mineralocorticoid (MC) receptor (MR) to varying degrees. In addition, there are numerous different routes of administration (including oral, intravenous, inhaled, and topical). There is therefore a vast spectrum of available drugs and formulations, and the precise choice of agent is determined by their clinical utility and efficacy but also balanced against the potential for adverse effects (19).

All steroidal synthetic GCs are structurally related to hydrocortisone (cortisol) but have often been modified such that they bind to the GR with a higher affinity and thus have more potent GC actions or have a prolonged half-life. Most synthetic GCs are 21-carbon polycyclic compounds with a basic steroid structure, a fused 17-carbon atom ring system with an aliphatic side chain at carbon-17, a keto-group at carbon-3, 1 or 2 double bonds in the A-ring, and an oxygen at carbon-11 (Fig. 1). Differences in GR and MR activation are determined by the side groups. GC potency is increased by an 11-hydroxy group, while MC potency is increased by a 9α or a 6α-fluoro substituent. In contrast, a hydrophilic substituent at position 16 decreases both MC and GC properties (20). Metabolism of GCs (both endogenous and synthetic) is a crucial determinant of their ability to activate both GR and MR. While there are multiple enzyme systems that are crucial in clearing GCs, including the A-ring reductases (5α- and 5β-reductases), CYP3A4, and the 20α- and 20β-hydroxysteroid dehydrogenases (21, 22), perhaps the most fundamentally important are the 11β-hydroxysteroid dehydrogenases (11βHSDs) (see 11β-Hydroxysteroid Dehydrogenase Type 1 Inhibition section), which regulate target cell GC availability through the dynamic interconversion of active (hydroxy) and the inactive (oxo) forms (17). The presence of an 11β-hydroxyl group is essential for the anti-inflammatory, immunosuppressive effects of GCs and for the sodium-retaining effects of MCs (23-25). Therefore, the reversible interconversion of the 11β-hydroxyl into the corresponding 11β-keto group plays a pivotal role in the efficacy of prescribed steroids.

**Figure 1.**
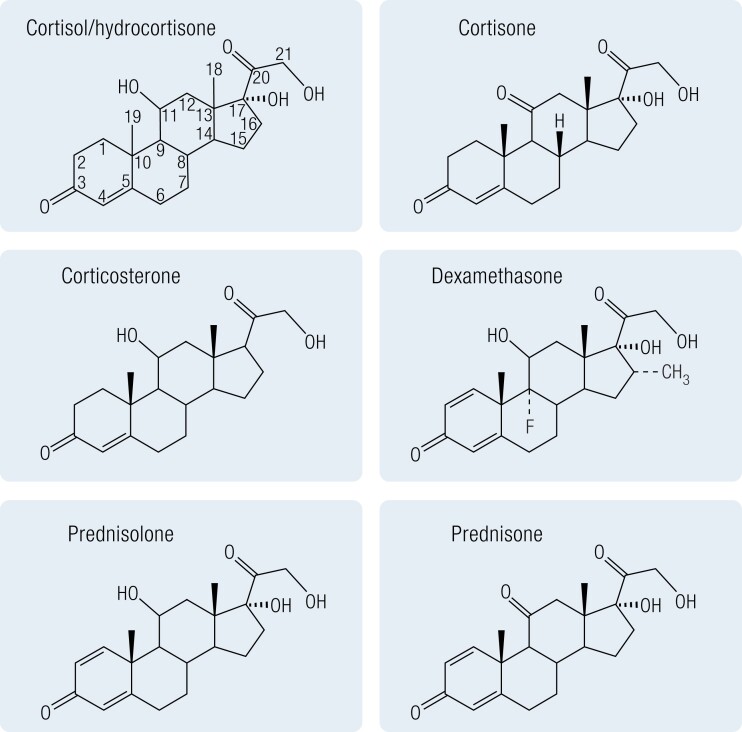
Chemical structure of endogenous and commonly prescribed glucocorticoids.

GC preparations have been developed to be delivered across multiple different routes of administration, including intravenous, intramuscular, oral, topical, inhaled, intranasal, and intra-articular use. While in the past it has been suggested that nonsystemic administration (topical, inhaled, intranasal, and intra-articular) might limit the incidence of system adverse effects, there is now good evidence documenting the systemic bioavailability (see also Table 2) and adverse effects (notably adrenal suppression) associated with administration of GCs via all routes (26-32). In particular, intra-articular administration and oral use have all been specifically associated with an increased risk of adverse effects (33). However, particularly in the treatment of inflammatory bowel diseases, second-generation oral corticosteroids (eg, beclomethasone dipropionate) have been designed to deliver topically acting corticosteroids directly to the site of inflammation (34, 35), reducing systemic bioavailability and some, but not all, adverse events (36, 37). Albeit promising, it is important to underline that these drugs are still absorbed systemically and are not free from steroid-related undesired effects and therefore should not be regarded as “steroid-sparing” agents yet.

**Table 2.** Systemic availability of different glucocorticoid formulations

Steroid formulation	Bioavailability	References
IV	>90%	561-563
Oral	76-99%	561-567
Nasal	First generation 40-50%	568
	Second generation 10-34%	
	Third generation <2%	
Inhaled	30-37%	569
Intra-articular	N/A	
Topical	N/A	

Abbreviations: IV, intravenous; N/A, not available.

Historically, the development of GC-induced side effects was thought to relate to both dose and duration of treatment with therapeutic benefit escalating in parallel with adverse effects (38-45). However, the precise association of dose and duration with increased risk of adverse effects is still not well established (40). Importantly, the severity of inflammation can be associated with both higher cumulative doses of GCs and systemic complications that may mimic the side effects attributed to GCs, and this cannot be resolved by statistical adjustments in observational studies (46).

A comprehensive review on the safety of prednisolone <10 mg/day (and often <7.5 mg/day) in patients with rheumatoid arthritis (RA) (39) collated data from multiple randomized controlled trials (RCTs) (47-55) and concluded that adverse effects associated with this treatment regimen were modest, and often not statistically different from those of placebo, with the exception of weight gain and glaucoma. However, relevant comprehensive adverse event data from published RCTs are still scarce. The relative safety of low-dose GC treatment does not appear to hold true for all GC-related adverse effects (56-59). Observational studies suggest a trend toward higher risks of cardiovascular disease, infection, diabetes, and higher mortality among patients taking GCs even at low doses (see also The Adverse Effects of Prescribed GCs section and Table 3) (60-65).

**Table 3.** Undesired effects of prescribed glucocorticoids related to dose and duration of treatment

	Dose related to increased risk	Duration of treatment/timepoint of evaluation
Mortality	>5 mg PE DD (171)	5 years
	>5 mg PE DD/ doubled risk each gram PE increase each year CD (58)	5 years
	>7.5 mg PE DD	6 years
	6% increased risk each gram PE increase in 1 year CD (164)	
	>8 mg PE DD	10 years
	>5 g PE per year CD	
	>40 g overall CD (63)	
	>5 mg PE DD (176)	13 years
	>7.5 mg PE DD (174)	15 years
Diabetes	>40 mg PE DD (210)	1 year
	1 mg fluticasone DD (207)	5 years
	>40 mg PE DD (205)	9 years
	60 mg PE IV single dose (67)	2 hours post GC dose
	60 mg PE IV single dose (66)	4 hours post GC dose
	>40 mg PE DD (70)	>2 days
	>7.5 mg PE DD for 2 weeks (57)	2 weeks
	>40 mg PE DD (211)	> 2 weeks
Hypertension	20 mg PE DD (224)	4 days
	>7.5 mg PE DD (41)	> 6 months
	>7.5 mg PE DD (43)	> 6 months
	>7.5 mg PE DD (238)	7 years
	>1 g progressively increase up to >3gr PE CD in the last year (58)	9 years
Osteoporosis/fractures	>15 mg PE DD	3 months
	>1 g PE CD (274)	
	>2.5 mg PE DD (59)	> 1 year
	>7.5 mg PE DD (248)	1 year
	>15 mg PE DD	7 years
	>5 g PE CD (273)	
Cardiovascular disease	>7.5 mg PE DD (308)	3 years
	>7.5 mg PE DD (307)	10 years
	>7.5 mg PE DD	<3 months
	>7 g PE CD (305)	
	>7.5 mg PE DD (315)	9 years
	>10 mg PE DD (317)	3 years
	>5 mg PE DD (319)	>1 year
	1-5 mg PE DD	1 to 10 years
	>1 g PE CD (320)	
Venous thromboembolism	>10 g PE CD overall (341)	6 years
	1-20 mg PE DD (9)	5-30 days
Cognitive dysfunction	>20 mg PE DD (347)	N/A
	>40 mg PE DD (356)	4 days
	>10 mg PE DD (358)	18 years
	>160 mg PE DD (360)	4 days
	>40 mg PE DD (364)	3-7 days
Infections	>10 mg PE DD (388)	>6 months
	>5 mg PE DD (389)	12 months
	>5 mg PE DD (391)	3 months
	>30 mg PE DD (391)	1 month
	>10 mg PE DD (392)	>15 days
	>5 mg PE DD (393)	7 years
	≤5 mg PE DD (394)	3 years
	≤5 mg PE DD (395)	N/A

Abbreviations: IV, intravenous; N/A, not available; PE, prednisolone equivalent; DD, daily dose; CD, cumulative dose.

The contribution of duration of GC treatment and its relationship with the development of side effects is also difficult to establish. Some studies have demonstrated harmful effects even after a single dose or brief treatment duration (66-72), and therefore clinicians will use therapeutic GCs in the lowest doses and for the shortest periods of time to achieve the desired therapeutic response.

Within this review, we discuss the clinical data that describes the breadth of adverse effects associated with GC therapy and explore the current and developing strategies to treat and limit adverse effects aiming to preserve the *good* and limit the *bad*.

## Classical Steroid Hormone Action

The GR is a ligand-activated transcription factor that resides in the cytoplasm. After ligand binding, the GR translocates, using the microtubule network, into the nucleus where it is able to enact various gene regulatory events. GR is a modular protein consisting of the standard nuclear receptor domains, an N-terminal domain, containing the activation function-1 domain, a DNA binding domain, hinge region, and ligand binding domain. Full exploration of the structure of GR is beyond the scope of this review and has been reviewed extensively elsewhere (73, 74).

Prior to activation, GR resides in the cytoplasm, held in a chaperone complex by HSP70 and HSP90, along with the immunophilin FKBP51. Binding of ligand to the ligand-binding domain results in a conformational change, exposing the nuclear localization sequence 1, driving the exclusion of HSP70, and a switching FKBP51 to FKBP52, which allows interaction with the dynein motor complex and active transport into the nucleus. Initially, HSP90 was also thought to dissociate; however, it now appears to be an important part of the translocation complex (75, 76). While translocation is an essential step along the path to activation of GR, it is not sufficient on its own to elicit a transcriptional response; the GR antagonist RU486 drives GR into the nucleus but does not permit gene regulation (77-79). Indeed, transport into the nucleus also can occur at a rate similar to that of diffusion, as has been demonstrated using nuclear localization sequence 1 mutants (80). This work identified a weaker sequence, NLS2, which signals for GR nuclear localization (not through a direct transportation process) but still results in nuclear accumulation, albeit at a much slower rate (80, 81).

Once ligand is bound, GR is phosphorylated at multiple sites (Ser203 and Ser211) by various kinases, including CDKs and MAPKs (82-84). Ser203 appears to be a preactivation step that must occur before the Ser211 phosphorylation, which confers full activation (85), and, as a result, Ser203 phosphorylated GR can be found in the cytoplasm, while Ser211 is almost exclusively nuclear (86). The phosphorylation sites of GR not only promote GR activity but can also be inhibitory; Ser226, phosphorylation via JNK, promotes GR nuclear exclusion and thus inhibits transcriptional activity (87). Different phosphorylation levels of GR have also been linked to differential transcriptional activity (88, 89) and its ability to recruit chromatin remodeling complexes (90). The respective functions of GR phosphorylation sites have been reviewed extensively (85), but there has been limited interest in exploiting differential phosphorylation patterns as a strategy to dissociate desirable and undesirable effects of GCs.

Once in the nucleus, GR binds to the DNA, detecting a cognate sequence in the major groove (91). The majority of GR DNA binding loci are via glucocorticoid response elements (GREs), palindromic DNA sequences containing the ACAnnnTGT core motif (92). The insertion of an engineered GRE sequence, proximal to the gene promoter, is sufficient to generate a GC-responsive gene, even in normally non-GC responsive genes (93), highlighting the importance of this sequence for GR activity. Small variations in the core motif sequence can have large effects on GR activity, even if the sequence modifications are in the spacer or surrounding sequence. This DNA-based modulation of GR thus adds a fine-tuning mechanism to the GC response at specific genes (94, 95).

Initial observations suggested that GR was, in the majority of cases, acting as a dimer on DNA binding (96, 97), with the dimer interface being important for GR activation (98). In recent work, the dimer configuration has been corroborated by more elaborate imaging- and sequencing-based techniques. Indeed, Presman and colleagues, using number and brightness assays, indicated that the majority of nuclear GR is in a dimeric form (99), while monomeric forms reside in the cytoplasm. However, others have highlighted that GR may form dimeric complexes in the cytoplasm (100) or without the necessity to bind to a GRE (101). Thus, it still remains an open question as to whether GR forms a dimer prior to DNA binding or dimerises on the DNA. It has also been suggested that a higher order quaternary structure of GR, going beyond the dimer, is possible, resulting in the formation of a tetramer (102), linking 4 GRs together as a “dimer of dimers.” A forced tetramer mutant (GRP481) was able to repress and activate more target genes and form more de novo open chromatin sites than wildtype GR (103), suggesting a biological role for the tetrameric form of GR. However, the exact mechanisms by which the putative GR tetramer works are yet to be elucidated.

The classical view of GR has been that the GR dimer is responsible for gene activation, while the monomeric form was rather involved in gene repression, through tethering to and inhibiting the action of other transcription factors, specifically AP-1 and NFκB (104, 105). Some work has suggested that GR may be mostly acting as a monomer (106); however, the prevailing view remains that it is the dimer that acts as the major factor in gene regulation. The tethering concept led to the development of the GRA46T mutant mouse, termed GRdim, which lacks dimerization capacity (107) and has been used extensively to study the dissociation of inflammatory repression by GCs from the metabolic actions of GCs, which were suggested to be dependent on dimer gene activation events. More recent phenotypic characterisation of these mice has demonstrated that the anti-inflammatory effects of GR are not entirely separable in terms of the monomeric/dimeric paradigm, in part due to the role of metabolism in preserving organismal function during severe disease and the requirement of GR to activate various anti-inflammatory genes (108-112). This view has now become relatively contentious, along with some criticism of GRdim as a dimer-deficient GR (99, 113). Genomic methodologies have identified GRdim preferring GR-half-site motifs (114). Reanalysis of this data using more defined GREs and GRE half-sites found that GRdim was not enriched for the half-site but rather a relaxed, degenerate GRE and suggested little to no physiological role for the monomer in regulating gene expression (113). This work, however, did not take into account ChIP-exo data whereby digestion of unbound DNA revealed GR-half-sites enriched in the GRdim cells (114). In contrast, a separate dimerization deficient mutant, the GRA477T mutant (GRmon), is capable of binding GR-half-site motifs on DNA and has diminished the ability to regulate gene expression (115). There is an excess of GREs in relation to GR-regulated genes, with many regulated genes having multiple functional GREs. In addition, there is a massive excess of GREs through the genome in relation to available GR protein molecules. Cell type specificity is conferred by lineage determining factors altering chromatin around such GREs to facilitate productive GR recruitment, a mechanism perhaps best explored in the liver, where the HNF4A factor plays a dominant role (116). Further questions therefore persist as to how, or if, monomeric GR is able to regulate gene expression.

### Transactivation

As a broad generalization, the gene regulation by GR can be broken down to transactivation, that is, the upregulation of gene expression through an intermediate protein, in this case GR, or transrepression, the suppression of gene expression again using an intermediate. While the 2 mechanisms share a similar intermediate, there is substantially more known about transactivation, while transrepression still remains somewhat underexplored. To activate genes, GR will bind to GREs and allow other regulatory factors to bind. For example, the Mediator complex components MED1 and MED14, both of which can directly interact with and assist in the initiation of RNA polymerase II activity (117), bind to GR, indicating a role for GR in the direct initiation of transcription (118, 119). Transcriptional control can also be influenced at the level of chromatin, not just the initiation complex. Here GR plays an important role in recruitment of chromatin remodeling enzymes that open the DNA and thus allow access for transcription to occur (120). In this context, GR can regulate the localization of the SWI/SNF complexes to the DNA, which in turn catalyse unpacking of chromatin to allow transcription to occur (90, 121-123). Indeed, interaction with such chromatin remodelers appears to be a crucial component of how GR activates gene expression, with methyltransferases [CARM1 (124), G9a (125), COMPASS complex (126) and histone acetyltransferases (CBP and p300) (127)] acting as major players driving gene expression.

### Transrepression

As described already, the traditional view of how GR inhibits gene expression was thought to be via protein-protein interactions and tethering to other transcription factors onto the DNA, preventing them from fully engaging the cofactors required for positive gene regulation. There are several key transcription factors that have been implicated, including AP-1, NFkB, and STAT3 (105, 128, 129). Similarly, a concept of specific DNA sequences containing negative GREs (nGRE) that result in gene repression rather than activation has been suggested. For example, these putative nGREs have been found in the POMC and CRH genes and have been suggested to be a major part of how GCs cause negative feedback on the hypothalamo–pituitary–adrenal (HPA) axis (130, 131). While it has been postulated that nGREs are found throughout the genome and represent a key determinant of GR-mediated gene repression (132), these findings have not been corroborated by cistromic studies (133, 134).

Major findings using genomic methods (including chromatin immunoprecipitation and sequencing) have identified that while GR and proinflammatory transcription factors can be found at the same genomic loci, binding to the DNA is a poor predictor of whether a gene will be up- or downregulated (134). More recent work has identified cryptic GR binding sites within NFkB and AP-1 response elements, adding a further layer of complexity as to how GR may be repressing gene expression (135, 136).

The recruitment of repression-specific cofactors also plays a core role in determining which genes are repressed by GR (134). Epigenetic state and chromatin condensation are also important mechanisms by which GR can repress gene expression. For example, recruitment of HDAC2 is necessary for efficient IL1β inhibition (137), and there is HDAC6-dependent repression of osteocalcin in determination of lineage commitment (138). HDAC3 is likely to be involved in GR repression through nGREs in a complex mechanism involving simulation of GR (139). There is also a concept that GR transactivation of repressors may provide a unifying mechanism to explain both gene transactivation and gene transrepression (140). This wide variety of mechanisms that GR seems to use to repress genes has made finding a core function relatively difficult and is currently a major focus of research.

### Nongenomic Actions of GR

Much attention has been focussed on the very potent effects of GCs that rely on transcription and then translation to have their effects (141). The nongenomic actions are often demonstrated using transcriptional inhibitors, such as cycloheximide, or by using the GR transcriptional antagonist, RU486, which allows translocation but not gene expression. These nongenomic effects are often attributed to interactions with kinases. GR activation promotes protein kinase A activation to regulate chloride release (142), AMPK and CAMKII inhibition to limit glucose uptake (143), p38 and JNK activation (144) and Akt, GSK3B, and mTOR activation to regulate proliferation (145). Recent work has also demonstrated that GR and RAS, an upstream regulator of MAPK signaling, interact with a potential role in cancer, linking many of these kinases to a central node (146). These rapid effects also contribute to the anti-inflammatory actions of GCs, especially in T-cells where GC treatment prevents phosphorylation of Lck and Fyn, limiting T-cell activation (147). However, kinases are not the only targets of nongenomic GR actions; other enzymes such as nitric oxide synthase have also been implicated as nongenomic GR targets. Activation of exhaled nitric oxides, although likely mediated via AKT, has been proposed as a mechanism by which GCs are able to rapidly aid in the treatment of heart attacks (148). GCs also provide rapid relief from the symptoms of asthma and are commonly used as inhaled therapy. They can cause rapid vasoconstriction by limiting noradrenergic signalling (149), prevent excessive inflammation by inhibition of T-cell function (147), and promote bronchodilation through cAMP signalling in bronchial smooth muscle cells (150). Interestingly, stimulation of cAMP signalling through nongenomic GR action at the membrane may also contribute in a limited way to the gene regulatory effects of GCs (151), putatively through recruitment of other cAMP-dependent transcription factors. Importantly, while many studies have investigated the nongenomic actions of endogenous GCs, synthetic GCs are also able to elicit nongenomic actions (152-154). Furthermore, there is some evidence to suggest that the hierarchy of potency of GCs to drive classic genomic GR activation may differ when compared to nongenomic actions (155). The precise contribution of nongenomic to the desirable and undesirable actions of glucocorticoids remains to be determined.

### GR Polymorphisms as a Determinant of the Response to Exogenous GCs

The ability of the GR to be activated by both endogenous and exogenous GCs provides a complex layer of regulation that has the potential to impact significantly on GC action (both desirable and undesirable). Genetic variations within the GR have been reported to be associated with a number of metabolic, cardiovascular, and inflammatory conditions and has been reviewed extensively elsewhere (156). The role of genetic variations to influence the therapeutic response, or the development of adverse effects in repines to GC treatment, has only been examined in a small number of studies. An asparagine-to-serine change at codon 363 with the GR (N363S) is associated with and enhanced sensitivity to exogenous GCs as demonstrated by greater suppression of endogenous cortisol and an exaggerated elevation in circulating insulin (157). Additional single nucleotide polymorphisms (including A829G and G459V) also appear to enhance transactivation potential (158, 159). Several splice variants of the GR have also been identified, and there is evidence to suggest that these may also have an augmented response to exogenous steroids (160).

## The Adverse Effects of Prescribed GCs

**Figure 2.**
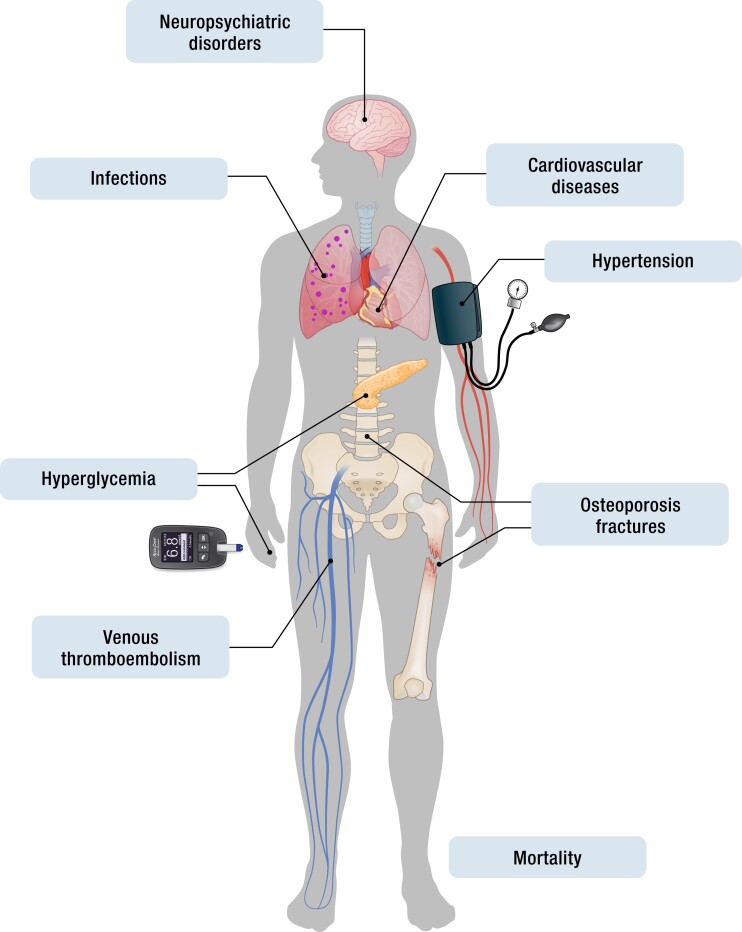
The side effect burden associated with prescribed glucocorticoids.

The clinical benefits of GCs are not in doubt. However, especially when used at high doses, for a prolonged period of time (>2 weeks), and in a reverse circadian regimen (higher doses in the evening compared to the morning), treatment is associated with significant adverse effects (17). These include, but are not limited to, increased overall mortality, metabolic effects [glucose intolerance or diabetes mellitus (DM), dyslipidemia], and musculoskeletal disorders (osteoporosis, osteonecrosis, myopathy, sarcopenia), as well as hypertension, adrenal suppression, and an increased risk of infection (161) (Fig. 2). The breadth and potential severity of this adverse effect profile often creates a challenging decision for both treating clinicians and patients who derive clinical benefit from GC treatment, while accepting the significant side-effects profile that may occur (161-163). Here, we summarize the published data detailing the adverse effects of GC therapy and outline the current management strategies that have been used.

### Mortality

Many of the side effects of the long-term GC use have been associated with a high risk of mortality (164). The majority of the published studies have largely focussed on all-cause mortality, although some have also specifically looked at cardiovascular mortality (60, 63, 165-168).

GCs have been associated with an increased risk of all-cause mortality in most (58, 60, 63, 166-171) but not in all studies (168, 172, 173). Similar inconsistencies have been documented for cardiovascular-specific mortality (63, 167, 168). Most of the studies demonstrated a direct relationship between GC dose and mortality, with 7.5 mg of prednisolone (or equivalent) being a reportedly “safe” threshold dose (60, 63) below which mortality was not increased (Table 3).

There are important methodological issues to consider when associations between GC exposure and mortality are examined, and true causal relationships cannot be demonstrated in these observational studies. First, it is not easy to unravel the complex relationships between the indication for treatment (and this may change over time) and the outcome. Also, the confounder of the specific disease indication of GC therapy needs to be considered. This is important bearing in mind that GCs are often prescribed to patients with advanced disease severity and at the end-of-life care. This so-called “perimortal bias” (164), where illness severity in the latter stages of life influences GC prescription ,is largely unaccounted for in the majority of published studies. Second, the large size of the populations needed to generate robust mortality data means that detailed information on duration and variability of GC dose (and consequently an assessment of total GC exposure) alongside all the other confounding variables can be lacking. Nonetheless, despite these limitations, important clinical messages still emerge.

In a prospective, 10-year follow-up study on 779 patients with RA, both all-cause and cardiovascular-related mortality were increased in GC users compared to nonusers, even when the data were corrected for the contribution of existing cardiovascular risk factors and RA disease activity and severity (63). Mortality risks increased in parallel with daily dose as well as cumulative dose: 8 mg/day and 5 g/year [prednisolone equivalent (PE)] were the treatment levels above which mortality rates increased (hazards ratio 1.22 to 2.11, respectively). These data were subsequently reproduced in a larger retrospective cohort (164). In this study, the authors included more than 16 000 patients with RA who were either never, previously, or currently treated with GCs. Previous or current GC use was associated with increased all-cause as well as cause-specific mortality with a largely consistent dose-response effect. In this analysis, when treatment doses were increased by 5 mg PE per day, there was an associated increase in the risk of death and a 6% increase in all-cause mortality for each gram (PE) increase in cumulative dose prescribed during the study timeframe. Taking doses less than 5 mg PE per day at the time of death did not increase the risk of all-cause or cause-specific mortality. Taking less than 7.5 mg PE per day at the time of death did not increase this risk of death from neoplasia or other noncardiovascular and nonrespiratory causes. Other published studies have also endorsed these data (171, 174).

A similar dose-dependent mortality risk was found in a further retrospective cohort of more than 70 000 patients with chronic autoimmune diseases treated with high doses of GC therapy. Higher mortality rates were observed in GC users when compared to patients who did not receive oral GC treatment at follow-up. In this study, mortality rates remained elevated in patients taking 5 mg PE per day, with further increases of about 6% for each increase of 5 mg PE per day and doubled risk for any gram PE increase in cumulative dose each year (58).

GCs are crucial as replacement therapy in primary and secondary adrenal insufficiency (15, 175). GC replacement doses used in patients with adrenal insufficiency are often significantly higher than estimated endogenous cortisol production rates in healthy subjects (10-15 mg/day) (176). Although causality cannot be established, it has been suggested that overtreatment with excessive GC replacement doses may contribute to adverse outcomes and increased mortality (177-180). Hammarstrand et al (176). examined the standardized mortality ratio in 392 patients with nonfunctioning pituitary adenomas in Sweden. Daily hydrocortisone replacement doses above 20 mg (or 0.30 mg/kg) were associated with increased mortality compared to patients with lower replacement doses and to those patients with an intact HPA axis. Patients with daily doses of ≤20 mg hydrocortisone had a mortality risk comparable to patients without GC replacement and to the general population. This study confirmed data from 2 previous reports demonstrating an increased mortality [relative risk 4.0 (178) and 3.79 (181), respectively] for patients receiving >30 mg hydrocortisone per day. Unfortunately, none of these studies reported analysis on cumulative dose.

### Diabetes

Both endogenous and exogenous GC excess impair glucose metabolism and predispose to diabetes through many complex pathophysiological mechanisms that are distinct from other causes. As a consequence, the American Diabetes Association has defined DM due to endogenous GC excess as a “specific type of diabetes secondary to endocrinopathy” (182).

There are several distinct, concurrent pathophysiological mechanisms that are responsible for GC-induced insulin resistance and the onset of DM in patients with GC excess (68, 72, 183, 184). Chronic hypercortisolism has direct effects on all major peripheral tissues responsible for glucose homeostasis including the liver, skeletal muscle, and adipose tissue. In the liver, GCs directly stimulate gluconeogenesis by the activation of 2 critical enzymes: phosphoenolpyruvate carboxykinase and glucose-6-phosphatase (185, 186).

GCs also act on other tissues shuttling gluconeogenic precursors to the liver, for example, by increasing lipolysis in white adipose tissue (187) and protein catabolism in skeletal muscle (188) and therefore driving glucose production and fueling the development of hepatic steatosis (189). The latter is worsened by GC-induced hepatic insulin resistance and the potentiated action of counterregulatory hormones, such as glucagon (190). GCs also inhibit the production and the secretion of insulin by the pancreatic beta cells (191).

Skeletal muscle is the primary site of insulin-stimulated glucose uptake and glycogen storage in the postprandial state (192). GCs directly disrupt insulin signaling in skeletal muscle by downregulating the expression and phosphorylation of IRS1, PI3K, and PKB–AKT (193-195). This results in concomitant inhibition of insulin-induced recruitment of glucose transporter 4 and its translocation to the cell surface of skeletal muscle (196), dampening skeletal muscle insulin sensitivity and glucose uptake, finally contributing to the development of GC-induced DM.

GC-induced DM is one of the most well-established adverse effects of hypercortisolism (197), although the prevalence is almost certainly underestimated (190) due to the heterogeneity of the published study populations and their different susceptibility to hyperglycemia. This is likely to reflect the differing indications for GC treatment and heterogeneity in age as well as comorbidities and underlying genetic predisposition. In addition, investigations to diagnose DM are not always performed during GC treatment. Fasting plasma glucose and insulin levels may not have enough sensitivity to assess glucose homeostasis in this context. More than half of patients with endogenous GC excess have apparently normal fasting glycemia (198) and oral glucose tolerance testing (OGTT) is not routinely performed (199). GC-induced hyperglycemia or DM is, however, a very common complication, and global estimates suggest that approximately 2% of new-onset cases of DM are linked to GC use (68, 72, 183, 200).

The crude incidence of GC-induced DM varies between 15% and 40% and is dependent upon the underlying indication for GC therapy including respiratory (201), renal disease (68), RA (202), solid organ transplant (203), or cancer (204) indications.

Two separate case-control analyses of registry data from the United Kingdom and New Jersey Medicaid, USA, have demonstrated increased odds ratios (OR) of 1.36 and 2.23 for development of new-onset DM due to GC therapy, respectively (200, 205). These data have been confirmed in a large meta-analysis of 6602 patients treated with GCs reporting an OR of 1.7 for new-onset DM (42).

GCs appear to exacerbate hyperglycemia in a dose-dependent manner irrespective of prior history of DM (205, 206); exposure to a single dose of exogenous GC can result in significant hyperglycemia in patients with or without pre-existing DM (66, 67). More than half of hospitalized patients without a history of DM experienced at least 1 episode of hyperglycaemia after GC treatment (70). When treatment is prolonged for more than 1 month, the incidence of GC-induced hyperglycemia and DM are 32% and 19%, respectively (71).

Overall, the detrimental effects on glycemic control are dose (205) and time dependent (70) (Table 3). When GCs are prescribed at high doses, all the common routes of administration (oral, topical, or inhaled) can cause hyperglycemia (205, 207-209), albeit the greatest risk is associated with oral formulations due to their higher systemic exposure (210). In patients with respiratory disease, high-dose inhaled GCs (1 mg of fluticasone equivalent per day) have been shown to increase the incidence of DM by approximately 34% (207). In a small study in patients with RA treated with prednisolone (5 to 15 mg per day), nearly 10% of patients developed DM within 2 years from the start of GC treatment (72). Unfortunately, a subgroup analysis examining the impact of GC dose was not performed. In a prospective study of patients with primary renal disease (but without DM) treated with prednisolone 40 mg/day, 42% of patients had 2-hour post-lunch glycaemia higher than 200 mg/dL (11.1 nmol/L) but normal fasting glucose levels (68). Similarly, 50% of patients receiving the same dose of prednisolone for the treatment of neurological conditions developed DM (211). In this study, fasting plasma glucose concentrations were consistently below 100 mg/dL (5.55 nmol/L) and therefore cannot be relied upon to confirm the diagnosis (212). A case-control study on patients receiving less than 40 mg/day (hydrocortisone-equivalent) showed a 2-fold increase risk for starting oral hypoglycemic therapy or insulin. The OR increased linearly with GC dose with a 10-fold increased risk in patients treated with more than 120 mg (hydrocortisone equivalent)/day (205). In a very small study, there is evidence to suggest that even low, supraphysiological doses of GCs (prednisolone 7.5 mg daily) can impair glucose tolerance with prolonged administration (57), although this has not been confirmed in larger cohorts.

Several studies have tried to dissect the risk factors that underpin the susceptibility to develop GC-induced DM. Age (greater than 60 years) (68, 201, 213), prior glucose intolerance/impaired fasting levels of glucose/insulin resistance (68, 214-217), being overweight (213), and elevated hemoglobin A1c levels (HbA1C) (>6%) (56) all appear to drive increased risk. A recent meta-analysis identified that the risk of GC-induced DM was greatest in subjects with abdominal adiposity and in those taking higher doses of GCs and for longer periods (70, 205). In a further study in patients with rheumatic or renal diseases receiving 40 mg of prednisolone daily, 66% developed GC-DM over a 4-week period. Higher HbA1c level (>6.0%, OR 3.05) and lower estimated glomerular filtration rate (<40 mL/min/1.73 m^2^, OR 3.42) were both independent risk factors for developing GC-DM (56).

In general, the effect of steroids is usually transient and reversible. GCs are likely to cause the greatest effects within the second and fourth week, but the effects can be seen even after few hours of administration during high-dose treatment (71). Thereafter, as GC dose is reduced, glucose metabolism often returns to pretreatment levels and GC-induced hyperglycaemia is expected to resolve (218, 219). However, this is not true in all cases (220, 221), and hyperglycaemia can persist up to 2 years after discontinuation, suggesting long-lasting detrimental effects on insulin sensitivity and β-cell dysfunction (222) or unmasking pre-existing glucose metabolism disorder (223).

### Hypertension

There is an established association between hypertension and the use of oral GCs (40). The risk factors predisposing to GC-induced hypertension are unclear and have not been examined in the same depth as GC-induced hyperglycemia. However, the duration of exposure and daily dosage are thought to be important (41-43), as well as a family history of essential hypertension (224).

The mechanisms underpinning GC-induced hypertension are complex and still not completely understood (225, 226). It has previously been thought that the MC effects of GCs were the main drivers to the development of hypertension. However, there is emerging evidence to suggest that this may not be the case. The MC action of most commonly prescribed synthetic glucocorticoids is much lower than that of endogenous cortisol. Furthermore, the administration of spironolactone, an MR antagonist, does not prevent or control GC-induced hypertension (227-229). Sodium intake also does not appear to contribute to blood pressure change in patients taking GCs (230-232). However, there is evidence supporting a role in GCs driving alterations in vasoactive substances impacting on the balance between vasoconstriction and vasodilation (including catecholamines, nitric oxide, and atrial natriuretic peptide) as well as activation of the renin-angiotensin system activation and causing cardiac hypercontractility (229, 233-236).

In a large meta-analysis of randomized, placebo-controlled trials, the risk of hypertension was found to be doubled in patients exposed to GCs when compared to those receiving placebo, regardless of the duration of exposure (42). There is considerable variability in the incidence of GC-induced hypertension compared with other known adverse effects. New-onset hypertension was observed in 9% of patients after 3 months of high-dose GC treatment (69). Incidence rates vary from 3% to 30% in patients with rheumatic conditions exposed to medium- to high-dose GCs (7.5-30 mg/day of PE) (40, 237). This rises to 37% in patients over the age of 65 years (224).

More recently, 2 large retrospective cohort studies have made significant contributions to the published literature. The first study included more than 70 000 patients with chronic inflammatory diseases. Compared to GC nonusers, there was a dose-dependent increase in the risk of hypertension in oral GC users (14%, 20%, and 30% for patients treated with <1 g, 1-3 g or >3 g of PE dose per year, respectively) (45). In the second study with >17 000 patients with RA but without hypertension at the time of diagnosis, GC use was associated with an 44% increased risk of hypertension (238). The risk remained increased (17%) even when correcting for age, sex, body mass index, smoking status, and the most common risk factors related to RA (such as serum uric acid). The risk was directly related to the GC dose, although at doses below 7.5 mg PE per day there was no increased risk (39, 43). These findings have been endorsed by some but not all studies (41) (Table 3).

### Osteoporosis

GC-induced osteoporosis (GIOP) is an important and common clinical problem and has been recognized ever since the first descriptions of endogenous GC excess (239). However, due to the widespread use of therapeutic GCs in the past 60 years, GIOP is now recognized as the most frequent cause of secondary osteoporosis worldwide (240, 241). It is well established that GC treatment is associated with significant loss of bone density, deterioration in bone structure, and significantly increased fracture risk (59, 242-245). The mechanisms underpinning GIOP are entirely distinct from those driving age-related or postmenopausal osteoporosis (246). Defining the mechanisms driving GIOP in vivo is complicated by the almost universal involvement of underlying, usually inflammatory, disease as the reason for initiating GC treatment in the first instance (246, 247). Inflammation itself, as well as underlying disease activity, may have substantial effects on bone, independent of GC use. As such, GCs, particularly when used at modest doses, can retain beneficial effects on bone through their anti-inflammatory actions (47). However, in some situations, there is still the potential that they may exacerbate bone damage through worsening the fine balance between bone formation and resorption. Increased fracture rates, even with daily doses as low as 2.5 mg PE, are reported (248). Evidence from mouse models suggests that the adverse effects of GCs on bone occur through direct actions on cells coordinating bone metabolism. Briefly, GCs increase osteocyte apoptosis and suppress osteoblasts activity and therefore limit bone formation (249) while increasing osteoclast-mediated bone resorption (250, 251). Impaired cellular proliferation and autophagy, increased apoptosis, changes in RANKL/osteoprotegerin, Wnt/sclerostin expression, and local active steroid availability through 11β-HSD1 activity have all been proposed as mediators of these effects (250, 252-255). Reduced bone formation at trabecular bone sites and increased endocortical resorption are the most consistent pathological findings. Details about all the mechanisms involved have been extensively reviewed elsewhere (256, 257).

Aside from the direct detrimental effects, GCs can also negatively impact on bone health by other indirect mechanisms; these include a reduction in sex steroid levels (258), reduced intestinal calcium absorption (259) with increased renal calcium excretion (260), altered PTH levels (261), adverse effects on muscle strength and consequently on bone strength (262), and a related increased risk of falls.

Fracture is the most common, serious, and preventable adverse event associated with GC use (263, 264). Up to 20% of patients develop an osteoporotic fracture (mostly commonly in the spine, proximal femur, and ribs) within the first year of GC treatment (265, 266); fracture risk is highest during the first few months of therapy and occurs independently from changes in bone mineral density (BMD) (248, 267, 268). The proportion of patients developing fractures rises to 50% after 5 to 10 years (242, 269), and fracture risk remains elevated for the duration of GC therapy. However, risk decreases significantly when treatment is ceased, although whether it returns to baseline values is unclear (267). The mechanisms for these rapid changes in fracture risk are not completely understood, but changes in bone density alone are unlikely to be the only explanation. An increased risk of falls due to proximal skeletal myopathy associated with GC use is also likely to be a major contributor (256).

Recent studies have confirmed the high predisposition to vertebral fracture in GC users, the dose-dependent increase in fracture risk, and decline in fracture risk with longer duration or discontinuation of GC therapy. Approximately 30% to 40% of patients on chronic GC treatment are diagnosed with fragility fractures (59, 244, 248, 270, 271). In patients initiated on GC therapy within the past 6 months, the annual incidence of vertebral fracture is 2-fold higher than at nonvertebral sites (244). Further insights into the prevalence of GIOP can be derived from the placebo arms of RCTs (265, 266, 272). A recent meta-regression of data from the placebo arms of these studies has reported annual incidence rates of vertebral fracture of 5.1% and 3.2% for patients initiating or continuing glucocorticoid treatment, respectively (244). The corresponding rates of nonvertebral fractures were 2.5% and 3.0%. Although fractures are most commonly seen at these sites, they can occur at virtually any skeletal site (59, 256).

There is also an additional dose and duration of treatment effect (244, 248, 273, 274) (Table 3). The effects of oral or injected GC therapy on fracture incidence rates are 3 times higher in those treated with doses >15 mg PE/day compared to those taking lower doses. However, even relatively modest doses of GCs have been associated with increased fracture risk (as low as 2.5 mg PE/day being linked to spine fractures). A fracture risk increase of 20% has been documented with a 5 mg prednisolone dose per day, increasing to 60% with a daily doses of 20 mg (59, 248, 256).

Multiple large population-based studies have independently shown that fracture risk decreases rapidly after GC exposure is stopped. Fracture risk is reduced by about 30% 6 months after stopping treatment (273, 275, 276) and normalizes by 12 months post-treatment (273).

The impact of treatment duration is often hard to disentangle from dose (248, 273). In a large population-based cohort study with more than 1200 patients, recent (<12 months) and prolonged (>3 months) GC exposure was associated with BMD-independent major fracture incidence (277). Other studies have provided reassurance that GC use is only harmful to bone health when used for relatively long durations.

However, a large population-based case-control Danish study with data from more than 81 000 patients demonstrated a significantly increased risk of fractures in heavy GC users (eg, >1 g PE cumulative dose) that was independent of the duration of treatment (274). Fracture risk did not seem to rise with higher doses, suggesting a clinical threshold of 1 g PE as cumulative dose threshold to guide clinicians in starting therapeutical prevention strategies.

Finally, there are conflicting data from studies evaluating the effects of pulsed GC therapy. A single study has demonstrated that intermittent use of high-dose GCs is relatively safe in terms of bone health (278), but this was not confirmed in other retrospective studies in which even short-term GC use (<30 days) was associated with an approximately 2-fold increase in fracture risk (9). As cumulative GC exposure associates with fracture risk, it is conceivable that even pulsed administration of GCs could be deleterious to skeletal health (241, 243).

When examining routes of administration, the negative skeletal effects of prolonged oral or intravenous GC treatment are well demonstrated (241). The impact of inhaled GCs on fracture risk remains unclear but is almost certainly confounded by intermittent oral (or parenteral) GC administration (279, 280). There are data to suggest that conventional inhaled GC treatment with modest doses for relatively short periods (<3 years) is not associated with bone loss or fractures (243, 281), However, an increase in fracture risk has been documented for high-dose (>600 μg/day dose of beclomethasone or equivalent), long-duration (>8 years), inhaled GC treatment (257, 282). While there are only limited data, topical steroids do not seem to be associated with increased fracture risk (243).

Additional data from patients taking GC replacement therapy have also contributed to our understanding of GIOP. Patients with Addison's disease treated with high-dose replacement have a time-dependent reduction in BMD at the spine and the hip (283). Importantly, recently evidence suggests that there is no reduction in BMD in patients with Addison's disease (or other causes of adrenal insufficiency) treated with lower GC replacement doses (284). However, not all data are consistent, and a higher prevalence of vertebral fractures (285) (30% vs 13%) has been documented in patients with Addison's disease despite no differences in BMD. Reducing GC replacement dose was efficacious in increasing spine and hip z-score, but the impact on fracture risk has not been examined. A degree of caution has to be used when interpreting data from patients with adrenal insufficiency because of the possible additive negative contribution of adrenal androgen deficiency (in Addison's disease) and a greater chance of coexisting other hormone deficiencies, for example in patients with hypopituitarism (286). Similarly, in patients with CAH, the cohorts are often heterogeneous, and there is the contribution of androgen excess alongside the impact of GC therapy (287-290). Overall, BMD appears to be reduced at all skeletal sites in CAH, and, as for patients with Addison's disease, this reduction correlates with cumulative GC exposure in some (289) but not all the studies (290).

Both under- and overtreatment might be responsible for the increased fracture risk seen in patients with adrenal insufficiency (256). A Swedish population-based analysis in more than 3000 patients demonstrated an approximately 2-fold higher risk of any fracture compared to age- and sex-matched controls (291, 292). The risk was 3 times higher in the year before the diagnosis was made, suggesting that GC deficiency may also have a major negative effect on bone (292).

Beyond reduced bone density leading to osteoporosis and fractures, a specific disease of femoral neck needs mention, namely steroid-induced osteonecrosis (SIO; also referred as “avascular” or “ischemic necrosis”). The risk of osteonecrosis with GC use is well established; however, it is often challenging to establish causality between GC use and osteonecrosis because many of the conditions that GCs are used to treat can also cause, or at least predispose to, osteonecrosis (293). According to the Association Research Circulation Osseous task force, the diagnosis of SIO can only be made in patients clinically diagnosed with osteonecrosis by symptoms, signs, imaging, and/or histological examination and with a history of systemic GC use >2 g of PE within a 3-month timeframe, a diagnosis made within 2 years after systemic GC use, and if all other risk factors besides systemic GC and other etiologies have been excluded (294).

The pathogenesis of SIO remains to be elucidated. However, the ultimate mechanism seems a compromised blood flow resulting in an inability to supply nutrients to crucial areas of bones, leading to bone death (295, 296). Several underlying factors are involved, including vascular damage, increased intraosseous pressure due to mechanical stress, adipocyte dysfunction, and defects in apoptosis and coagulation (295). Early diagnosis is essential as prognosis and treatment options decline as the disease progresses.

After trauma, GC use is the second most common cause of osteonecrosis, with a prevalence ranging between 3% and 40% (297, 298). Approximately two-thirds of patients with SIO are younger than 60 years old with an average age in the early 30s and a male prevalence (293, 299).

Due to the contribution of the underlying different diseases for which GC are used, it has been a challenge to determine whether the risk of SIO is more closely related to cumulative dose, maximum dose, or duration of therapy, and currently there are no guidelines that define a safe threshold dose of steroids (293). In general, a GC dose greater than 30 mg of PE daily or a cumulative dose ≥12 g PE per year cause a significant increase of osteonecrosis risk. This is even higher with longer half-life GC formulations and if the cumulative dose exceeds 5 g of PE within the first 3 months (293). However, there are reports describing SIO with a much lower dose of GC (295). Unfortunately, bone resorption and formation markers seem not to be helpful in predicting SIO development (300).

With respect to the route of GC administration, SIO is commonly associated with parenteral or oral GC (293, 295). However, patients who receive GC via other routes (such as intra-articular, inhaled, intranasal, topical) have low, but not negligible, risk of developing SIO (293, 298, 301-304), although these studies have the main confounder of concomitant treatment also with other oral or parenteral steroids. Patients should always be informed of the risk of developing osteonecrosis whenever GCs are used.

### Cardiovascular Disease

Whilst the anti-inflammatory and immune-suppressive effects of GCs can have beneficial effects to limit the atherosclerotic and vascular injury related to chronic inflammation (305), prolonged GC can increase incident cardiovascular disease (CVD), including heart disease and stroke (306-308). The mechanisms by which GCs increase CVD risk are a composite of fueling the development of adverse metabolic syndrome features (including central adiposity, hyperlipidemia, insulin resistance, and hyperglycemia) (309-311) alongside mineralocorticoid effects (including cellular membrane electrolyte-mediated efflux) (312, 313).

In a cohort study including approximately 70 000 patients exposed to more than 7.5 mg PE per day, the risk of unfavorable cardiovascular outcomes (defined as a composite end point of hospitalization with a primary diagnosis of myocardial infarction, angina, angioplasty or coronary revascularization, stroke, transient ischemic attack, congestive cardiac failure, or cardiovascular death) was 2 to 4 times higher compared a similar number of unexposed patients (308). Interestingly, the increased risk of coronary heart disease and heart failure was higher than that of stroke (307, 308).

While several, mainly retrospective, studies have demonstrated that current GC use is associated with increased risk of composite CVD, coronary heart disease, myocardial infarction, heart failure, stroke, and atrial fibrillation (305, 307, 308, 312, 314-318), the impact of prior GC use has not been systematically evaluated. In addition, there is evidence for a dose-dependency of effect on CVD risk and data suggesting that a low daily dose of prednisolone (5 mg or less) may be safe and associated with only a limited risk (307, 308, 315, 319) (Table 3). However, more recent data in patients without previously known CVD prior to GC prescription would appear to contradict these observations (320). The authors assessed the incidence of 6 common CVDs (atrial fibrillation, heart failure, myocardial infarction, stroke, peripheral arterial disease, and abdominal aortic aneurysm) and confirmed that the risk of developing CVD was dose and duration dependent (307, 308, 317). However, even at low GC doses (5 mg PE/day), CVD risk was 2-fold higher than before treatment (6-fold higher in those on 25 mg PE/day or more). The CVD risk associated with high-dose GC treatment was similar to that seen in patients with diabetes or established CVD.

### Venous Thromboembolism and Pulmonary Embolism

Whether GC treatment conveys a thromboembolic disease risk remains contentious. GCs are widely used for the treatment of many inflammatory diseases, and inflammation itself can modulate hemostasis (321, 322).

An increased incidence of venous thromboembolism has been described for several inflammatory diseases, including asthma (323, 324), chronic obstructive pulmonary disease (325, 326), inflammatory bowel disease (327-329), arthritis (330-332), and other autoimmune diseases (333, 334).

It is therefore hard to separate the potential prothrombotic effects of GCs from the hypercoagulable inflammatory state (335-337). However, 2 studies including healthy participants treated with oral GCs compared to placebo demonstrated a GC-related risk (338, 339).

GC can target several components of the coagulation cascade and act through different mechanisms (340). Dexamethasone increased circulating levels of several coagulation factors including FVII, FVIII, FXI, and fibrinogen (339). Similar results were found following a 10-day course of prednisolone, which induced a procoagulable state by increasing thrombin generation, as well as plasminogen activator inhibitor-1 and von Willebrand factor levels (339). Furthermore, a sensitivity analysis on a systematic review on 23 studies investigating GC treatment in a setting of acute inflammation demonstrated that GCs caused significant inhibition of fibrinolytic activity. Of note, the effects of GC might be different whether they are administered in the context of inflammatory or noninflammatory disorders. Other additional mechanisms to mention include reduction in activated partial thromboplastin time, rise in platelets, thromboxane B2, thrombin-antithrombin complexes and fibrinogen levels, and, importantly, impaired fibrinolytic capacity (340).

Data from the UK have suggested a 3-fold increased risk of venous thromboembolism/PE in current users of oral GCs compared with nonusers. The risk was higher in current users (OR 4.68) and decreased with treatment duration, but a 2-fold increase in risk persisted where treatment lasted more than 1 year (341). Subsequent studies have confirmed these data and suggested that the risk occurs across differing routes of administration (including inhaled therapy), is highest among new users, and may be associated with dose-dependent cumulative GC exposure (341-343) (interrater reliability 1.4 to 2.27, respectively) (9) (Table 3).

### Mental Health and Cognition

Chronic exposure to supraphysiological doses of GC dose is associated with anatomical brain changes and an increased prevalence of psychiatric disease, cognitive impairment, mood alterations, and sleep disturbance (344-346). Adverse psychiatric side effects occur frequently during exogenous GC treatment. Psychosis, mania/hypomania, depression, and anxiety are the most common findings, with a prevalence ranging from 3% to 60% of cases (347). Cognitive impairment is also frequently observed, and up to 7% of patients complain of long-lasting cognitive deficits following GC treatment (348).

Neurocognitive decline associated with exogenous GC use is typically characterized by euphoria (349) with deficits in declarative and working memory, mental processing speed, and concentration (350). Both short-term (351, 352) and long-term (353) GC treatment can adversely affect memory performance (354, 355) with a more significant decline in elderly patients (354, 356). In addition, there appears to be a GC dose dependency of effects (347, 356-364).

There is no agreed consensus as to the degree of the reversibility of mental health and cognitive impairments following GC withdrawal. Several reports have documented complete cognitive recovery within weeks of GC discontinuation (365, 366), but long-term (up to 1 year) persistence of mild cognitive impairment has also been described (351, 367) (Table 3). In addition, there is evidence of improvement in brain atrophy following GC withdrawal (368-371). We have recently extensively reviewed elsewhere the contribution of GC on cognitive function (372).

### Infection Risk

GCs have multiple inhibitory effects on immune cell number and function (16, 373, 374) leading to overall immune suppression characterized by cell death, impaired immune regulation, and defective immune response (375). A comprehensive analysis of their impact is beyond the scope of the current review and has been extensively reviewed elsewhere (375-378).

Briefly, GC dampen neutrophil response to inflammatory stimuli (379), increase susceptibility to viral as well as opportunistic infections inducing lymphopenia (mainly of the CD4 + subset) by altering CD4/CD8 (380) and Th1/Th2 ratio subpopulations (381), suppress cytotoxic activity in natural killer cells (382), reduce eosinophil count (383), and reduce mast-cells interfering with IgE-mediated mast-cell degranulation and calcium release (384). Interestingly, some of these effects seem to be dose dependent and different in exogenous vs endogenous GC excess (375).

Due to the multitude and complexity of quantitative and qualitative immunosuppressive effects that arise as a result of GC therapy, their use is associated with an immediate increase in the risk of infection, especially with common bacterial, viral, and fungal pathogens (385-387).

Infection risk is directly related to GC dose as well as the duration of treatment (Table 3); long-term (>6 months) use with doses >5 mg PE/day are associated with a >2-fold increase in the risk of serious infection (388-393). However, several retrospective studies have suggested that lower doses (≤5 mg PE/day) are also associated with a clinically meaningful increase in serious infection risk (388, 390, 394), especially when given for a prolonged duration (>3 months) (391).

However, there are some conflicting data. A meta-analysis of 21 RCTs demonstrated no increase in risk, but event rates were very low, and the studies were underpowered to detect differences in serious infection risk (395). In the same study, a meta-analysis of observational studies (>50 000 patients) demonstrated a dose-dependent increased risk of infection starting from a relative risk of 1.37 for those under 5 mg PE/day dose up to the 2-fold risk associated with higher doses (395). Importantly dose- and duration-dependent risks fell sharply within 6 months of discontinuing GC treatment (391). Finally, patient-specific factors may influence infection risk; older age, lower functional status, and underlying disease activity as well as other concomitant immune-suppressive drug administration all are associated with higher infection risk (396-398).

## Strategies to Ameliorate the Adverse Effects of Prescribed GCs

### Conventional Comorbidity Management

#### GC-induced hyperglycaemia

The monitoring and management of GC-induced hyperglycaemia (GIH) is a very common clinical challenge facing physicians across all specialities, and there are sparse data from observational and interventional trials upon which to guide therapy and no clear established therapeutic goals or targets (399).

The glucose threshold at which hypoglyeemic treatment should be started is also not clear. Some authors suggest that an implementation of glucose-lowering therapy should be started when pre- or post-prandial glucose repeatedly exceed 140 mg/dL (7.8 mmol/L) or 200 mg/dL (11.1 mmol/L), respectively (399-401), and similar treatment strategies used in patients with T2D, including a stepwise intensification of anti-hyperglycemic therapy as well as frequent re-evaluation. The American Diabetes Association suggests using similar targets as for other types of diabetes, aiming to individualize glycemic control according to patient-specific factors including life expectancy, comorbidities, compliance, and risk of hypoglycemia (402). In general, in hospitalized critically and noncritically ill patients, a target glucose range of 140 to 180 mg/dL (7.8-10.0 mmol/L) is recommended, although tighter control may be appropriate for selected patients, if this achievable without increasing the risk of hypoglycemia (400). There are specific patient populations (eg, short life expectancy due to incurable disease, advanced age, and comorbidities) in which the best practice will be the avoidance of both hypo- and hyperglycemia (400, 402, 403). Importantly, after GC treatment has been initiated, there are frequent dose adjustments that must be made, matching the dose adjustment in GC dose with glucose-lowering therapies.

Lifestyle modifications (hypocaloric diet and adequate low-moderate physical activity) are currently recommended for high-risk subjects predisposed to DM, but it should be recommended for all patients undergoing treatment with GCs (404). Some oral antidiabetic drugs have demonstrated the potential to improve glycemic control and prevent or delay the development of GIH (405, 406). First-line treatment should potentially include drugs that increase insulin sensitivity, targeting postprandial hyperglycemia as well as the other adverse effects of GCs, such as weight gain, increased fracture risk, and cardiovascular events such as metformin and pioglitazone (407-410), which can be also continued in patients with pre-existing T2D unless there are contraindications.

Two small RCTs have demonstrated that in patients initiated on a mean daily dose of 30 mg of prednisone equivalent, metformin therapy prevented the rise in plasma glucose after 4 weeks of treatment (407) as well as improving HbA1c levels, pancreatic beta cell function, and insulin sensitivity after 12 weeks (409).

Thiazolidinediones act on both hepatic and peripheral insulin sensitivity, targeting 2 of the major metabolic undesired effects of GCs (411). However, the evidence base for the use of pioglitazone over and above other treatments is relatively weak (408). Pioglitazone improved glycemic control in prednisone-treated patients in a small study (412). However, it takes several weeks to have its maximal effect, and the treatment-related adverse effects (such as weight gain, increased fracture risk, fluid retention, and, importantly, heart failure) may potentially exacerbate the adverse effects of GCs (411, 413-417). For this reason, pioglitazone is less attractive and perhaps is not regarded as a first-line therapeutic option in patients with GIH. Similarly, there is evidence for the beneficial effects of insulin secretagogues on GIH (418, 419), but their use can be compromised by weight gain, hypoglycemia, and a possible increased cardiovascular risk (413, 420). Insulin secretagogues can be used to treat mild GIH in the inpatient setting, specifically in nonseverely ill patients who receive short-acting steroids once daily in the morning (400).

The mechanism of action of glucagon-like peptide-1 (GLP-1) analogues and dipeptidyl peptidase-4 inhibitors is predominantly to lower postprandial glucose, and as a result they have potential utility to combat prednisolone-induced hyperglycaemia. Intravenous exenatide improved blood glucose levels in healthy subjects taking 2 days of high-dose prednisolone (406). Unfortunately, longer term data on the efficacy of GLP-1 agonists in GIH are lacking, but their benefit in reducing cardiovascular risk is a potential added benefit. Due to their gastrointestinal adverse effects profile, the use of GLP1-receptor agonists cannot be the first choice for acutely ill, hospitalized patients with GIH (399).

The dipeptidyl peptidase-4 inhibitor sitagliptin improved glycaemic control in a retrospective study of subjects on low-dose prednisolone (421) but was not able to prevent the diabetogenic effects of a 30 mg daily prednisolone dose in a prospective RCT (405).

Sodium-glucose co-transporter-2 inhibitors reduce postprandial glucose in patients with T2D. Concerns regarding use include the risk of euglycemic diabetic ketoacidosis (particularly among patients with poor food intake) and the risk of genitourinary infections (422), albeit the benefits of this class of drugs seem to outweigh these risks of infection (423, 424). While the use of the sodium-glucose co-transporter-2 inhibitor dapagliflozin has shown safety in patients hospitalized for chronic obstructive pulmonary disease developing GIH, it did not improve glycemic control or clinical outcome (425).

Glucose-lowering therapy in hospitalized patients often requires rapid control of glucose elevations, and therefore the Endocrine Society Clinical Practice Guidelines recommend subcutaneous basal bolus insulin as the treatment of choice for GIH in the hospital setting. Continuous insulin infusion can be considered for severe or persistent hyperglycemia (426). When insulin is used in both the hospitalized and nonhospitalized setting, it is important to match the pharmacokinetics of the GC preparation with the precise insulin regimen in order to optimize glucose control and reduce the risk of hypoglycemia.

#### Hypertension

The clinical monitoring of patients prescribed with exogenous steroids should include blood pressure measurements performed on a regular basis. Currently, there is no universal consensus about the treatment of exogenous glucocorticoid-induced hypertension, and, due to the lack of dedicated studies, evidence is derived mainly from patients with endogenous hypercortisolism. However, considering the pathophysiology similarities and currently available guidelines, extrapolations to exogenous GC administration can be made (427, 428). In this regard, a recent consensus of the working group on endocrine hypertension of the European Society of Hypertension has been released (428). Lifestyle interventions with the aim of reducing cardiovascular risk, including diet, diet, physical activity, weight normalization, and cessation of smoking, must be encouraged (240). Efforts should be made to use the lowest needed dose of GCs, but often pharmacological measures to treat hypertension are necessary. Angiotensin-converting enzyme inhibitors as well as angiotensin receptor blockers are often considered as first-line approaches to lower blood pressure due to their cardioprotective effects (226, 429, 430). When compared to other antihypertensive drugs, calcium channel blockers have demonstrated high efficacy in reducing blood pressure levels and reducing cardiovascular events, both alone and in combination with angiotensin-converting enzyme inhibitors/angiotensin receptor blockers (226, 428, 429, 430). They are often considered as second-line agents. If the addition of calcium channel blockers is ineffective or contraindicated, mineralocorticoid receptor antagonists might be considered as a third- or second-line treatment especially in those cases with coexisting hypokalemia (430, 431). Considering the hypogonadism and gynecomastia that can be associated with spironolactone treatment (432, 433), eplerenone can be considered as an alternative (226). A possible new attractive therapy concerns Finerenone, a nonsteroidal mineralocorticoid-receptor antagonist. However, it has demonstrated only modest effects on blood pressure reduction (434), lower efficacy, and risk of adverse events including hyperkalaemia when compared to spironolactone (435), and, therefore, it might not be an alternative option. Currently there are no data on its use during exogenous GC treatment.

Alpha blockers and nitric oxcide donors might be also considered as additional therapies in those uncontrolled with conventional therapies (226). Beta-blockers and thiazide diuretics should be used with caution; both may have unfavorable effects on glucose metabolism, and thiazide may worsen pre-existing hypokalemia (430, 436-438). However, in selected cases, such as those patients with previous myocardial infarction, vasodilating beta-blockers (including carvedilol, labetalol, and nebivolol) may be considered, as benefits to improve cardiovascular risk related to myocardial infarction might outweigh other risks (437, 439). A similar risk-benefit analysis can be applied to hydrochlorothiazide, which may be considered in those patients with kidney stones due to its effect in preventing cortisol-induced hypercalciuria (430, 440).

#### Osteoporosis

Although recent guidelines differ in the precise indications for pharmacological intervention, in general, patients planning to start GC treatment for more than 3 months at any dose and who have experienced a prior fragility fracture should start bone protective therapy, regardless of age or GC dose (386, 441-444). It is recommended that this should be initiated at the same time as GC treatment. Due to the reversibility of the effects of GC on bone, all current treatment guidelines suggest that bone protective therapy can be stopped after GC therapy is discontinued unless there is an additional prior indication (443). However, epidemiologic studies published after the guidelines were released suggest that there may be an increase in fracture risk that persists for up to 15 months after GC treatment has been stopped (274, 276, 278). Clinicians may therefore choose to continue therapy for another 3 to 6 months for lower cumulative exposures (less than 1 g. of prednisone equivalent cumulative dose) or longer periods (6-18 months after GC discontinuation) for higher cumulative exposures (275).

Although evidence is limited, a number of nonpharmacological measures and lifestyle modifications may mitigate the harmful skeletal effects of GCs, and it remains important to maintain the lowest GC dose needed to control the underlying inflammatory disease (241). Similar to other forms of osteoporosis, vitamin D and calcium supplements are generally advocated to increase BMD (445, 446) but, as per nonpharmacological measures, their effects on fracture risk remain unclear (447).

Bisphosphonate therapy is regarded as the first-line option for GIOP (448). Bisphosphonates bind to the bone surface and reduce bone turnover by inducing apoptosis in actively resorbing osteoclasts, which in turn leads to a reduction in osteoblast activity (449). In addition, inhibition of osteoblast and osteocyte apoptosis may also contribute to their mechanism of action in GIOP (450).

Alendronate, risedronate, and intravenous zoledronate are the most widely used agents and are all approved for GIOP (451). All have been shown to increase BMD at the spine and hip in GIOP (266, 272, 452-456) Furthermore, in post hoc analyses in patients receiving moderate to high doses of GCs, alendronate, risedronate, and ibandronate (452, 455-457) have shown a reduction in vertebral fracture incidence. The data for nonvertebral fracture are less clear with benefit in some studies but not all (243, 458-460).

Denosumab, a human monoclonal antibody directed against RANKL (a master activator of osteoclast differentiation), with a potent inhibitory antiresorptive effect on bone, has also been used for GIOP. In recent large double-blind, active-controlled RCTs in patients receiving GCs (≥7.5 mg PE/day), denosumab was superior to risedronate in increasing BMD (461, 462). However, no differences were found in the occurrence of fractures between treatment arms.

The pathophysiology of GIOP with impairment of osteoblast-driven bone formation provides a strong rationale for the use of anabolic agents. Such agents have the potential to restore both bone quality and quantity when compared with bone resorption inhibitors, especially in those patients with prolonged exposure to high doses of GCs (463). The only osteoanabolic agent that has been studied in RCTs in GIOP is teriparatide, the recombinant 34 amino acid peptide of PTH. In the first double-blind RCT evaluating the efficacy of teriparatide vs alendronate in 428 patients with osteoporosis who had received ≥5 mg PE/day for >3 months, the increase in BMD was higher in the teriparatide-treated patients after 6 months of therapy (464). Similar data have been observed in other studies with an additional decrease in vertebral fractures (465-467).

A recent Cochrane review (459) and 2 double-blind RCTs (461, 464) have demonstrated the effectiveness of bisphosphonates, teriparatide, and denosumab to treat GIOP. As a result of these and other studies, oral bisphosphonates (alendronate, risedronate, and ibandronate) are recommended as first-line agents to prevent GC-related fractures, while intravenous bisphosphonates (zoledronate and ibandronate), subcutaneous teriparatide, and subcutaneous denosumab are recommended as second-line agents (264).

The use of newer osteoanabolic agents such as abaloparatide (a synthetic analogue PTH-related peptide) (468) and romosozumab (a monoclonal antibody against sclerostin and acting on the canonical Wnt/β-catenin signaling pathway) (469) have not been evaluated in GIOP (470). Similarly, estrogen replacement therapy alone, combined hormone replacement therapy with progestins, or selective estrogen receptor modulators have not been thoroughly evaluated in the management of GIOP (471, 472) and their use is restricted to selected cases. A recent meta-analysis of RCTs evaluating the effects of testosterone replacement therapy on different bone parameters showed that testosterone improves BMD at the lumbar spine only in hypogonadal patients: the effect was greater in subjects with lower testosterone levels at baseline and increased as a function of treatment duration (473). These data endorse the use of testosterone to improve BMD only in patients with gonadal insufficiency.

### Selective GR Agonists and Selective GR Modulators

The precedent for an approach to selectively modulate steroid hormone action in a tissue-specific manner has been set through the development and clinical use of selective estrogen receptor modulators. These drugs are able to act as estrogen agonists and antagonists, depending on the target tissue, and are now established in clinical practice for management of breast cancer, osteoporosis, and post-menopausal symptoms (474). Adopting a similar approach aiming to separate the anti-inflammatory action of GCs has been challenging, and progress into the clinic has been slow (475-477). This is a reflection of the complex nature of GC intercellular signaling, including an imperfect understanding of how to distinguish transactivation and transrepression (189, 478).

#### Mechanisms of selective GR agonists action

**Figure 3.**
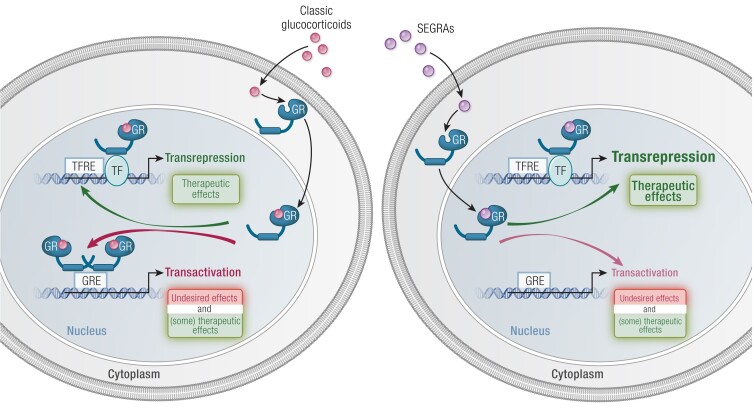
Genomic effects of classic glucocorticoids (left) and Selective Glucocorticoid Receptor Agonists (SEGRAs, right).

While GCs are extremely potent for their intended target, as discussed earlier, they are associated with debilitating side effects (see The Adverse Effects of Prescribed GCs section). Therefore, substantial work has aimed to develop so-called selective glucocorticoid receptor agonists (SEGRAs) in order to try to separate the negative side effects from the beneficial anti-inflammatory actions (Fig. 3). This was often based on the idea that a monomer/dimer dichotomy existed regarding repression/activation. However, as discussed in the Classical Steroid Hormone Action section, this model of GC action is no longer clear-cut; monomers have been found to activate and not simply repress gene expression (114), and also to activate anti-inflammatory genes that are required for the full effects of GCs to act as anti-inflammatory agents (111, 479, 480). Early reports identified compound A as a selective agonist that seemed to have limited ability to activate genes via GR but efficiently repressed genes involved in inflammation (481). However, recent work highlighted that the differential effects of compound A are likely through alternative cofactor recruitment (482) rather than an effect on tethering, and data supporting selective binding of compound A to the GR is unconvincing.

#### Preclinical studies

Early enthusiasm for pursuing selective GR ligands was based on the idea that changes in ligand structure could result in selective activation of transactivation or transrepression. A large number of candidate compounds were advanced to preclinical studies in animal models based on screening of compounds against a transactivation assay, thought to be the mechanism for metabolic side effects, and transrepression of NFkB, thought to be the key mechanism for anti-inflammatory action (483). These compounds failed to advance to clinical benefit, in most part due to a lack of robust separation of actions. This led to a revised view of how the activated GR can both activate and repress gene expression and how this might be achieved (116). It now emerges that there may be a common mechanism requiring GR to transactivate target gene expression and that the gene products of this conserved mechanism then drive anti-inflammation as a secondary effect (140). Indeed, candidate mechanisms involving phosphatases and other inhibitory molecules have been characterized (484).

#### Clinical studies

Among the SEGRA molecules that have been identified and studied in the preclinical setting, only a few have progressed into clinical trials.

ZK245186 is a nonsteroidal, topical, selective GR agonist (mapracorat, BOL-303242-X) (485) and has shown in vitro and in vivo anti-inflammatory actions (cytokine secretion and T-cell proliferation inhibition and a mechanism of action similar to methylprednisolone) and reduced adverse effects compared to classical GCs (with regard to induction of skin atrophy after long-term topical application, thymocyte apoptosis, and hyperglycaemic effects) in rats and, for this reason, was tested in several clinical trials to investigate its efficacy and safety for the treatment of atopic dermatitis and ocular diseases (allergic conjunctivitis, inflammation after cataract surgery). However, the search for these trial results (https://clinicaltrials.gov/ct2/results?cond=&term=Mapracorat&cntry=&state=&city=&dist=) identifies 13 studies, all showing the status “completed” between 2013 and 2020, but the full publication of study results is not yet in the public domain.

Two novel experimental GR agonists, AZD5423 and AZD7594, have entered clinical trials in the treatment of patients with asthma. AZD7594 improved lung function, asthma control, and symptoms and reduced airway inflammation in mild to moderate asthma, with only a mild impact on systemic markers of GC activity (osteocalcin and dehydroepiandrosterone sulfate. However, its efficacy was not compared against standard GCs therapies that are currently used to treat asthma (486-488). The efficacy of AZD5423 (at doses that have been shown to be efficacious in allergen-induced asthma) was tested against budesonide (at a dose that is twice that which is commonly used to treat the condition) in a large (n = 353), double-blind, randomized, parallel-group study. While AZD5423 showed a good side-effect profile (suppression of dehydroepiandrosterone sulfate but not cortisol or osteocalcin when compared to budesonide), neither AZD5423 nor budesonide had any clinically meaningful effect regarding lung function or markers of inflammation (489). Whether the results were affected by a selection bias is not clear, and further studies are needed to unravel the efficacy of AZD5423.

Fosdagrocorat (PF-4171327) is an orally administered partial GR agonist that has been assessed in 2 phase II trials for treatment of patients with RA. In the first, a multicenter, randomized, double-blind, parallel-group, active- and placebo-controlled trial, 86 patients with RA and methotrexate therapy were randomized to fosdagrocorat, prednisone, or placebo. Fosdagrocorat (25 mg) was superior to prednisone in the reduction of the disease activity score and C-reactive protein levels. While it caused more suppression of cortisol when compared to prednisone, the effects on osteocalcin levels were similar. However, fosdagrocorat did not cause lasting HPA axis suppression, and plasma cortisol concentrations returned to baseline values within 4 weeks of the last drug dose (490).

Subsequently, in a 12-week, phase II, controlled trial in 323 patients with RA randomized to once-daily fosdagrocorat (1 mg, 5 mg, 10 mg, or 15 mg), prednisone (5 mg or 10 mg) or placebo, fosdagrocorat (10 and 15 mg) showed noninferior anti-inflammatory effects compared to prednisone 10 mg. Nonsignificant improvements in glycaemic control and bone formation biomarkers were reported with equipotent anti-inflammatory effects in patients treated with fosdagrocorat (477). However, the development of this compound has been discontinued for undisclosed reasons.

More recently, the results of a novel nonsteroidal selective GR modulators, AZD9567, have been published (491). Two phase I trials were designed to assess AZD9567 safety, pharmacokinetics, and pharmacodynamics. Secondary outcomes for both studies included the effect of AZD9567 on glucose homoeostasis as measured with an OGTT. Exploratory outcomes aimed to test the effects of AZD9567 on inflammation (lipopolysaccharide-stimulated TNFα and inflammatory cytokines response in whole blood cells), HPA axis activity (plasma cortisol or 24 hour urinary cortisol), and on biomarkers of bone turnover (osteocalcin). Prednisolone was used as the comparator. The first trial was a randomized, placebo-controlled, single-blind, single-ascending dose study done in 72 healthy men, whereas the second was a randomized, active-controlled (prednisolone), single-blind, multiple-ascending dose trial in 75 healthy men and healthy women. AZD9567 and prednisolone exerted similar and potent anti-inflammatory effects. However, prednisolone 20 mg and 40 mg caused a substantial increase in plasma glucose across the OGTT, whereas AZD9567 (at an equipotent anti-inflammatory dose) had only minimal impact on glucose tolerance (similar to that of 5 mg of prednisolone). In vitro studies confirmed that AZD9567 did not upregulate the expression of genes involved in gluconeogenesis (tyrosine aminotransferase, phosphoenolpyruvate carboxykinase, and glucose 6-phosphatase), whereas prednisolone caused a significant induction of these genes. Plasma osteocalcin and cortisol levels decreased dose dependently after both the AZD9567 and prednisolone treatment to similar levels. AZD9567 was well tolerated; no serious adverse events were described, and plasma cortisol concentrations in all participants returned to normal levels 2 weeks from the last dose of study drug administration (491). Results from a phase II trial examining the effect of AZD9567 on glycaemic control in patients with T2D (NCT04556760) (https://ClinicalTrials.gov/show/NCT04556760) are awaited.

### 11β-Hydroxysteroid Dehydrogenase Type 1 Inhibition

**Figure 4.**
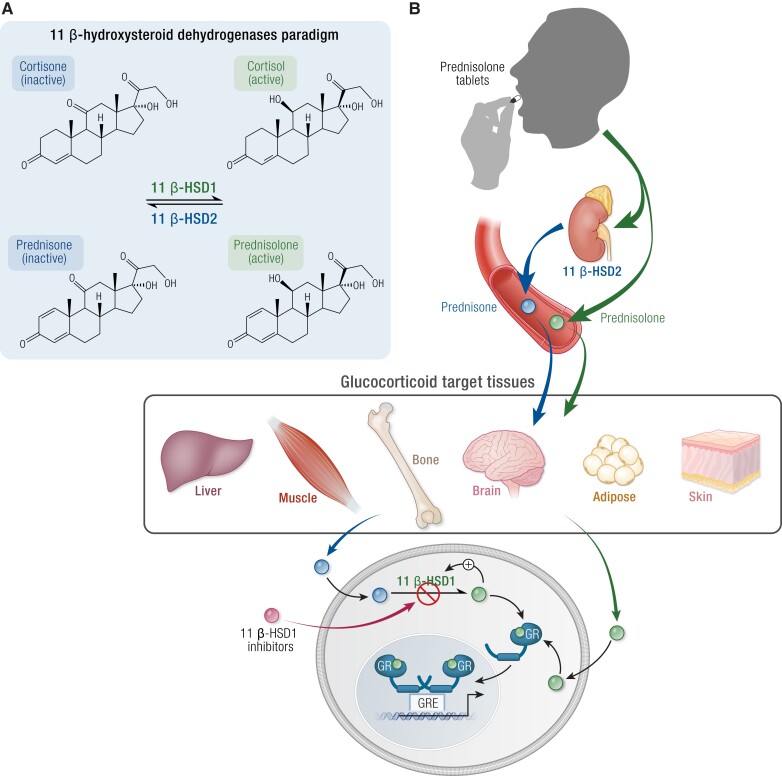
The 11β-hydroxysteroid dehydrogenases interconvert active and inactive glucocorticoids (A) and putatively have a critical role in regulating tissue-specific adverse effects that may be limited by 11β-HSD1 inhibition (B).

11β-hydroxysteroid dehydrogenases (11β-HSD) are enzymes that interconvert active and inactive GCs (24, 25, 492) and play a fundamental role in governing access for GCs to bind and activate the GR (Fig. 4). There are 2 isoforms that have been identified. 11β-HSD type 1 (11β-HSD1) interconverts the inactive GC, cortisone, and its active form, cortisol. It is widely expressed and, although bidirectional, in vivo it functions predominantly as an reductase, generating active GC (cortisol in humans and corticosterone in rodents) and therefore has the potential to amplify local GC action. Importantly, GCs also increase the expression and activity of 11β-HSD1, and therefore there is an additional “feed-forward” mechanism that further fuels GC availability and action within tissues (493). 11β-HSD1 is highly expressed in many tissues throughout the body including the liver, which has the highest level of expression, adipose (subcutaneous and intrabdominal), muscle, skin, brain, and bone (25). Patients with mutations in the gene encoding 11β-HSD1 (HSD11B1), or its accessory enzyme hexose-6-phosphate dehydrogenase (H6PDH) that generates its cosubstrate (NADPH) to permit reductase activity (25), develop cortisone reductase deficiency and apparent cortisone reductase deficiency, respectively (494-498). Both conditions are rare and present with a clinical phenotype resembling polycystic ovary syndrome with HPA axis activation (as a consequence of decreased cortisol regeneration), increased ACTH, and adrenal androgen excess.

In contrast, 11β-HSD type 2 (11β-HSD2) is principally expressed in mineralocorticoid target tissue including the kidney, salivary gland, and placenta. It is an nicotinamide adenine dinucleotide dependent dehydrogenase that inactivates GCs converting cortisone to inactive cortisone (492, 499). Patients with mutations in HSD11B2 present with the syndrome of apparent mineralocorticoid excess whereby, as a consequence of the inability to clear cortisol locally (principally within the kidney), it is able to bind and activate the MR for which it shares the same affinity as aldosterone, resulting in severe hypertension with life-threatening hypokalemia (500).

Importantly, both 11β-HSD1 and 2 are able to metabolize the synthetic GC prednisolone (interconverting active prednisolone with inactive prednisone) with similar kinetics as observed with cortisol (500). Dexamethasone is not metabolized in the same way; indeed, there is evidence that 11β-HSD2 activity may enhance the activity of dexamethasone (501).

Dysregulation of 11β-HSD1 activity has been suggested as a crucial mechanism unpinning the predisposition and development of metabolic disease including obesity, insulin resistance, and T2D (24). In addition, it may also have a role in the development of ocular hypertension, idiopathic intracranial hypertension, osteoporosis, and cognitive decline as well as part of the ageing process (252, 502-509). Based on this evidence, many selective and potent 11β-HSD1 inhibitors (selective in their ability to inhibit 11β-HSD1 and not 11β-HSD2) have been developed over the past 20 years and trialed in differing clinical contexts. While modest improvements in glucose control in patients with diabetes, lipid profiles, blood pressure, weight, intracranial pressure (in patients with IIH), hepatic steatosis, and wound healing issues have been observed (510-518), the magnitude of response is perhaps less than might have been expected based upon extrapolations from rodent models. The precise reason as to the relatively limited clinical impact is not entirely clear. However, it is plausible that this may be due to the activation of the HPA axis that occurs following 11β-HSD1 inhibition. Treatment with these drugs, in almost all studies, results in activation of the HPA axis as a result of impaired peripheral cortisol regeneration. There is a rise in ACTH, which increases adrenal cortisol secretion in an attempt to maintain eucortisolemia. It is this increase in cortisol production that has the potential to offset the beneficial effects of 11β-HSD1 inhibition.

There is now an emerging body of evidence to suggest that 11β-HSD1 may have a role to govern local GC action and the consequent development of the Cushing's phenotype in situations of circulating GC excess. The first indication of this potential role was highlighted through independently published case reports of Cushing's syndrome where the classical phenotype was absent (519, 520). The first was a case of biochemically and histologically proven Cushing's disease in which there was decreased conversion of cortisone to cortisol in comparison with age- and sex-matched controls, alongside a decreased cortisol half-life that is consistent with decreased 11β-HSD1 activity (519). The second case was one of Cushing's syndrome due to an adrenal adenoma, and again there was deceased cortisol generation and shortened cortisol half-life (520). In both cases, urinary steroid metabolite analysis used to assess 11β-HSD1 activity showed a profound decrease both in comparison to patients with known Cushing's syndrome (where activity is usually increased) and to healthy controls. The lack of clinical phenotype was therefore explained by a functional deficit in 11β-HSD1 activity (no genetic mutations were identified in either case) and an inability to regenerate active cortisol in key GC target tissues. Extending these observations, 11β-HSD1 knockout mice have been shown to be resistant to the development of a classical Cushing's phenotype in liver, muscle adipose, skin, and bone when high-dose corticosterone (the major circulating GC in rodents) was administered in the drinking water compared to their wildtype littermates (521, 522). Furthermore, evidence from 11β-HSD1 adipose tissue specific knockout mice, as well as mice with adipose tissue specific deletion of the GR, suggest that it is adipose tissue specific GC action that has a critical role in driving the adverse effects of exogenously administered GCs (521, 523). The combination of the clinical case reports alongside the preclinical data have raised the intriguing possibility that targeting 11β-HSD1 activity may represent a viable therapeutic strategy to limit the adverse effects of exogenous and endogenous glucocorticoid excess.

A single uncontrolled open-label study has tested this hypothesis in humans in which patients with endogenous GC excess (from a heterogeneous group of causes) were treated with a selective 11β-HSD1 inhibitor for 24 weeks. The results were variable and perhaps need to be interpreted with caution bearing in mind the limitations of the study design. However, there were beneficial changes in body composition with increased muscle mass and decreased fat mass (524). Additional placebo-controlled studies targeting 11β-HSD1 activity are now actively recruiting in the setting of endogenous Cushing's syndrome (525, 526).

More recently, we have presented data demonstrating that, in the context of exogenous GC excess (prednisolone 20 mg for 1 week), there are significant reductions in the adverse effects when this was combined with a selective 11β-HSD1 inhibitor (AZD4017), including glucose handling, adipose tissue lipolysis, circulating lipid profiles, and, perhaps most impressively, markers of bone turnover. It is important to question as to why the data from studies in GC excess models may be more conclusive than those in other disease contexts. Although not established beyond doubt, it is possible that this may reflect the lack of an intact HPA axis in conditions of GC excess. In exogenous GC excess and adrenal Cushing's, ACTH is suppressed, and in Cushing's disease, there is autonomous ACTH production that is not under the normal control of a negative feedback loop. As a result, there is no consequent activation of the HPA axis that can successfully reestablish tissue cortisol levels (527).

There are many questions that remain. In the context of exogenous GCs, these are often administered for their anti-inflammatory action. While our own data would suggest that the majority of these may be preserved, this study was conducted in healthy male individuals as a proof-of-concept with no underlying inflammatory conditions. The relative preservation of anti-inflammatory actions could potentially be explained by the tissue distribution of 11β-HSD1; it is highly expressed in liver, fat, muscle, skin, and bone with significantly lower expression in immune-inflammatory cells. Rodent data have also perhaps added a note of caution. 11β-HSD1 knockout mice do develop a more florid phenotype when challenged with specific inflammatory stimuli (528). In addition, the response of these conditions to GC therapy in some but not all aspects is blunted in the 11β-HSD1 knockout mice (529). However, the muscle loss associated with GC treatment of chronic inflammation was improved (530). Pharmacological inhibition of 11β-HSD1 in mouse models of inflammatory conditions treated with GCs has not been performed. Taking into account the well-described differences in GC metabolism and the effects of 11β-HSD1 deletion/inhibition between mice and humans, the clinical significance of the observations in rodents is yet to be determined but should not detract from the need to undertake dedicated clinical studies. Such studies are planned and currently in the set-up phase (531).

### Other Novel Potential Strategies

There are other potential targets that are as yet to be fully exploited in terms of their potential to dissociate the desirable from the undesirable effects of GCs. These data are confined to preclinical models, and clinical studies have not been performed. Elevated GCs levels have been associated with enhanced generation of 5-hydroxytryptamine (5-HT) and signalling through its receptors including the 5-HT2A receptor (532). In rodent models, 5HT2A receptor antagonism has been shown to ameliorate some of the metabolic phenotype associated with dexamethasone administration in adipose, liver, and skeletal muscle (533). However, signaling via the 5-HT2A receptor has been shown to have potent anti-inflammatory actions in rodent models (534, 535). It therefore remains to be determined if targeting 5-HT2A receptors could prevent adverse effects without compromising anti-inflammatory GC actions.

The liver X receptors (LXRα and LXRβ) are critical in regulating multiple metabolic and inflammatory pathways. While LXRα is almost exclusively expressed in the liver, LXRβ is expressed more widely. The data with regards to LXR and its role in inflammation are complex and sometimes contradictory, and there are studies showing that LXR activation can worsen (536, 537) or improve (538) inflammation. While rodent models have suggested that specifically antagonizing LXRβ may prevent some of the adverse metabolic side effects associated with GC use, with preservation of inflammatory actions, currently clinical data are lacking (539, 540).

### Chronopharmacology

GCs are crucial mediators of the interaction between the central and peripheral clocks to synchronize bodily functions. During exogenous GC treatment, the use of long-acting formulations, supraphysiological dose, or reverse circadian regimens disrupt clock synchronization caused by altering the cortisol circadian rhythm (17). This plays a significant role in the development of GC-related undesired effects (541-544). For these reasons, greater attention should be paid to the administration time of GC (eg, chronopharmacology), which could help restore physiological cortisol circadian rhythms, thus reducing the undesired effects. This can be accomplished by administering conventional GC at an appropriate time (therefore taking account of their pharmacokinetics, avoiding exposure to GC late in the evening and at night) or using special drug delivery systems (chronoformulations) to synchronize GC concentration to circadian rhythms in disease activity (545).

In the context of adrenal insufficiency, the development of modified-release hydrocortisone formulations (better mimicking the physiological cortisol daily profile) demonstrated efficacy in reducing metabolic disturbances caused by the peak and trough cortisol fluctuations seen during multiple daily doses of the conventional regimens. Indeed, switching to modified-release hydrocortisone formulation (Plenadren) has improved body mass index and body weight (546-548), glucose metabolism (548, 549), and quality of life (546, 548) and restored the immune alterations observed in these patients (548) to levels close to healthy controls.

However, the same approach is difficult to translate to the much more complicated pathogenesis of autoimmune and inflammatory diseases. In RA, for example, the circadian rhythm of symptoms, disability, underlying inflammation, and cytokine concentrations are well-known phenomena (550), usually with maximum severity in the early morning hours (551, 552) and preceded by elevated levels of proinflammatory cytokines (553). These findings led to the development of a new modified-release prednisone tablet formulation, which allows timed release of glucocorticoids 4 hours after oral administration (554), therefore acting on the nocturnal rise of proinflammatory cytokines when taken at bedtime. The 2 RCTs performed in patients with RA demonstrated clinical and biochemical efficacy of modified-release prednisone (554, 555) with good safety profile compared to immediate-release prednisolone regarding HPA axis function (556). The same concept was used to target the early morning rise in ACTH and 17-OH progesterone (17OHP) and replicate the overnight diurnal rise in cortisol in the context of CAH (557). In the phase 2 study, the delayed-release hydrocortisone compound (Efmody—development name Chronocort) demonstrated good disease control at a lower dose compared to standard treatment (558). Despite significant improvement of the clinical relevant endpoint (morning biochemical control of 17OHP and androstenedione), reduction in total daily dose and patients’ reported outcomes, the phase 3 study (559) failed to meet its primary endpoint (change from baseline to 24 weeks in the mean of the 24-hour 17OHP), probably for the prespecified methods for data analysis (560). Further studies are needed to confirm the superiority of the delayed-release formulation against current GC strategies.

## Conclusion

The burden associated with the adverse effects of GC treatment remains a major unmet clinical need. The use of GCs continues to rise as a result of their undoubted therapeutic efficacy and lack of alternative treatments. Strategies to combat their adverse effects have made relatively slow progress, despite the clinical need. This is almost certainly a reflection of the highly challenging and complex nature of the problem to ameliorate the “bad” without compromise to the “good.” There is some promise in the development of SEGRAs/ selective glucocorticoid receptor modulators and perhaps 11β-HSD1 inhibitors, but further development of novel compounds and approaches, alongside carefully constructed and controlled clinical studies are urgently needed.

## Disclosures

J.W.T. has been an advisory board member for Novo Nordisk, Pfizer, Poxel Diurnal, and Lumos; R.P. and G.C. have nothing to disclose; D.W.R. receives research funding from NovoNordisk and BristolMyersSquibb.
